# Identification of the First CHeRI Orbivirus 3–5 Strain Isolated from a Dead Farmed White-Tailed Deer (*Odocoileus virginianus)* Whose Death Had Been Attributed to an Infection by Mule Deerpox Virus

**DOI:** 10.3390/v18030305

**Published:** 2026-02-28

**Authors:** Emily DeRuyter, Pacharapong Khrongsee, Kuttichantran Subramaniam, Kristen Wilson, An-Chi Cheng, Zoe S. White, Amira Richardson, Merrie P. Urban, Juan M. Campos Krauer, Samantha M. Wisely, John A. Lednicky

**Affiliations:** 1 Department of Environmental and Global Health, College of Public Health and Health Professions, University of Florida, Gainesville, FL 32610, USA; emilyderuyter@ufl.edu; 2 Emerging Pathogens Institute, University of Florida, Gainesville, FL 32610, USA; firstpachar@ufl.edu (P.K.); kuttichantran@ufl.edu (K.S.); jmcampos@ufl.edu (J.M.C.K.); wisely@ufl.edu (S.M.W.); 3 Department of Infectious Diseases and Immunology, College of Veterinary Medicine, University of Florida, Gainesville, FL 32608, USA; 4 Faculty of Veterinary Science, Prince of Songkla University, Songkhla 90110, Thailand; 5 Department of Wildlife Ecology and Conservation, Institute of Food and Agricultural Sciences, University of Florida, Gainesville, FL 32611, USA; knwilson@ufl.edu (K.W.); zoezy5@gmail.com (Z.S.W.); 6 Department of Large Animal Clinical Sciences, College of Veterinary Medicine, University of Florida, Gainesville, FL 32608, USA; anc907@pitt.edu (A.-C.C.); amiramarierichardson@gmail.com (A.R.); merrie.urban1@gmail.com (M.P.U.)

**Keywords:** CHeRI orbivirus, mule deerpox virus, deer farming, white-tailed deer, Florida

## Abstract

We report the isolation and coding complete genome sequences of a new CHeRI orbivirus from the spleen of a dead farmed white-tailed deer in Florida whose death was attributed to an infection by mule deerpox virus. Phylogenetic and genetic analyses support this new virus as the fifth strain of the CHeRI orbivirus 3 species, and we designated it CHeRI orbivirus 3–5. While our previous detections and isolations of CHeRI orbiviruses were from deer spleens that also contained epizootic hemorrhagic disease virus-2, or in one case, Hardee County ephemerovirus 1, no deerpox virus was isolated from the spleen of the animal in this report, marking the first time we have isolated a CHeRI orbivirus without a co-infecting agent.

## 1. Introduction

The population of farmed deer in the United States has steadily increased, comprising approximately 287,000 animals as of 2022, the last year for which an estimate was available [1]. The state of Florida has an estimated 140 deer farms and 11,000 farmed deer, consisting primarily of white-tailed deer (*Odocoileus virginianus*, hereafter WTD) [1]. The University of Florida Cervidae Health Research Initiative (CHeRI) collaborates with WTD-farming stakeholders of Florida to help increase the health and sustainable production of captive cervids, as well as the native wildlife and ecosystems in which they live [2].

Farmed and wild deer within Florida can develop serious illnesses caused by arthropod-borne pathogenic bacteria, parasites, and viruses. Orbiviruses (order *Reovirales*, family *Sedoreoviridae*, genus *Orbivirus*) [3] are among the arthropod-borne viruses (arboviruses) that can cause serious illness in farmed and wild populations of WTD in North America and elsewhere [4,5,6]. They contain a segmented genome that consists of ten linear double-stranded RNA genomic segments of varying lengths. One copy of each genomic segment is packaged into each icosahedral orbivirus virion. The ten genomic segments are designated seg-1 to seg-10 and encode seven structural (VP1 to VP7) and four non-structural proteins (NS1, NS2, NS3/NS3a, and NS4).

As for other viruses with segmented genomes, two different orbivirus strains can exchange genomic segments if they co-infect a single host cell in a process termed reassortment. This process generates novel orbivirus strains with new properties that may include altered host range, increased virulence/pathogenicity, and immune evasion. Factors that may drive orbivirus reassortment include inadequate immunization, improper biosecurity practices, mixed farming practices, weather, vector competence and trade [5,7,8,9,10,11]. Infections within ruminants can results in a wide spectrum of clinical outcomes, ranging from subclinical or inapparent disease to severe illness characterized by high morbidity and mortality [12]. Experimental infections of WTD with BTV resulted in endothelial cell hypertrophy, thrombosis, hemorrhage, and vessel rupture; similarly, EHDV infections can result in hemorrhagic disease [13,14]. Variation in the clinical severity can be influenced by several factors. Our group identified age as one such determinant: in an assessment of BTV and EHDV prevalence among farmed WTD in Florida, the highest number of reported fatalities occurred in yearling animals [15]. Orbivirus outbreaks are of significant concern to farmed WTD stakeholders due to significant economic loss associated with increased abortions, morbidity, mortality, stillbirths, and restriction of trade [4,16]. Orbiviruses can replicate and be transmitted by arthropod vectors such as biting midges, mosquitoes, sandflies, and ticks [3,17]. Historically, BTV and EHDV outbreaks were limited to the home range of Culicoides midges, between latitudes 35° S and 40° N, but changes in the global range of the vectors have allowed for the occurrence of the disease between latitudes 35 °S and 50 °N [4,17,18].

Scientists affiliated with CHeRI discovered a new type of orbivirus in WTD, CHeRI orbivirus, which groups into four genetic lineages: CHeRI orbiviruses 1 through 4 [19,20,21]. They also found Big Cypress, Mobuck, and Yunnan orbiviruses in dead farmed WTD, suggesting that these viruses may also be significant pathogens of this cervid species [19,20,21]. Whereas it is known that BTV and EHDV are transmitted by biting midges of the genus Culicoides [4,22], the vector of CHeRI orbiviruses has not yet been identified. Emerging evidence suggests that mosquitos may serve as vectors of some orbiviruses, as two of them (Mobuck virus and Kevo orbivirus) were recently identified in Finnish *Ochlerotatus communis* mosquitos [23]. These observations highlight patterns that warrant further investigation into the potential role of mosquitos in the transmission of CHeRI orbiviruses.

Mule deerpox virus (DPV) was first identified in a cervid within the state of Florida in a dead WTD fawn in 2016 [24]. CHeRI researchers recently extended the initial finding by reporting that DPV affects deer throughout Florida [25]. In the work described below, DPV was isolated in Vero E6 (African green monkey kidney) cells from a skin lesion of animal OV1859. However, whereas the animal’s spleen tested positive for DPV by PCR, the virus was not isolated in Vero E6 cells inoculated with a homogenate prepared from that tissue. Instead, a different virus was isolated from the spleen homogenate in C6/36 cells, an *Aedes albopictus* mosquito cell line. Since DPV is not known to complete its life cycle in C6/36 cells, we suspected that a different virus had been isolated. Next-generation sequencing was thus performed on nucleic acids extracted from spent culture media from the C6/36 cells inoculated with spleen homogenate from animal OV1859, leading to the identification of the virus as a CHeRI orbivirus. The ability of the CHeRI orbivirus we isolated in this work to complete its life cycle in C6/36 but not in Vero E6 cells is consistent with our previous observations regarding other CHeRI orbiviruses [19,21,23]. Phylogenetic and genetic analyses supported the new virus isolate as a distinct and thus the fifth strain of the CHeRI orbivirus 3 species, and we designated it CHeRI orbivirus 3–5. The findings of this report exemplify the need for continued pathogen surveillance to identify viruses previously not described in farmed and wild WTD.

## 2. Materials and Methods

### 2.1. Animal History and Specimens Collected for Diagnostic Tests

The animal of this report (animal ID: OV1859) was included in a previous study on the epidemiology of DPV [25]. On 21 July 2023, a 2-month-old male farmed WTD from Jefferson County, Florida, USA, was observed lying in a lateral recumbent position and pawing the ground. The animal was treated with dexamethasone and banamine but was found dead two hours post-administration of the anti-inflammatory drugs. A field necropsy was performed on-site on the following day, 22 July 2023, revealing lesions consistent with DPV infection on one ear and a hoof. However, ulcers in the rumen and abomasum and lesions in the kidney and lung tissues were inconsistent with those expected for DPV but were consistent with those observed in hemorrhagic diseases caused by orbiviruses. Samples of skin lesion (LE) and tissues: liver (hepatic; HT), grossly normal skin (SK), spleen (ST), lungs (LT), heart (cardiac; CT), kidney (KT) and gastrointestinal tract (GI) were obtained in approximately 1 cm^3^ pieces and placed into 5 mL sterile Eppendorf snap cap tubes (Thermo Fisher Scientific, Waltham, MA, USA). Swab specimens of the lesion (LS), nasal cavity (NS), and feces (FS) were also obtained and placed in 5 mL tubes. Whole blood (WB) was collected via a cardiac puncture with a disposable 18 G needle and a 10 mL syringe (EXELINT International, Redondo Beach, CA, USA). The blood sample was transferred into 2 mL BD Vacutainer EDTA tube (Becton Dickinson, Franklin Lakes, NJ, USA) immediately after collection. All swab and tissue specimens and the whole-blood specimen were refrigerated during transportation and stored in a −80 °C freezer immediately upon arrival of the necropsy team at the UF College of Veterinary Medicine for virology tests at a later time.

### 2.2. Preliminary Diagnostic Assessment

DNA for DPV PCR tests was extracted from material extruded onto virus transport medium from swab specimens and from homogenized tissue specimens using a DNeasy Blood and Tissue Kit (Qiagen, Germantown, MD, USA) [25]. For virus isolation attempts, 10% (wt/vol) homogenates of the tissue specimens were generated in advanced Dulbecco’s modified Eagle’s medium (Invitrogen Corp., Carlsbad, CA, USA, Thermo Fisher Scientific, Waltham, MA, USA) supplemented with 2 mM L-alanyl-L-glutamine (GlutaMAX, Invitrogen Corp.) and antibiotics (50 µg/mL penicillin, 50 µg/mL streptomycin, and 100 µg/mL neomycin [PSN; Invitrogen Corp.]) using a manual tissue grinder (Covidien, Mansfield, MA, USA). RNA was extracted from the WB sample and from the spleen tissue homogenate using a QIAamp Viral RNA Mini kit (Qiagen) following the manufacturer’s protocol [21]. Conventional PCR for DPV was conducted using the protocol described in [25]. Additionally, due to the necropsy findings suggestive of a hemorrhagic etiology not consistent with DPV, multiplex reverse-transcription qPCR (RT-qPCR) targeting BTV, bovine viral diarrhea virus (BVDV), and EHDV was performed as previously described [21]. Cq values of 36 and under were considered positive, 37–39 suspect, and 40 and above negative. Total RNA purified from the tissue homogenates tested negative for BTV, BVDV, and EHDV (Table 1). The samples were subsequently tested using a one-step multiplex qPCR tests for CHeRI orbiviruses 1-3, Mobuck virus (MOV), Big Cypress orbivirus (BCOV), and Yunnan orbivirus (YUOV), as previously described [21].

### 2.3. Cell Cultures

Virus isolation was attempted in Vero E6 (*Cercopithecus aethiops* [African green monkey]) (Cat. no. CRL1586) and C6/36 cells (*Aedes albopictus* [Asian tiger mosquito]) (Cat. no. CRL1660) obtained from the American Type Culture Collection (ATCC, Manassas, VA, USA), as described in [21]. The cells were propagated as monolayers in 25 cm^2^ vented tissue culture flasks (25 cm^2^ flask, Corning Inc., Corning, NY, USA) using Advanced Dulbecco’s Modified Eagle’s Medium (aDMEM, Invitrogen Corp. Thermo Fisher Scientific, Waltham, MA, USA) supplemented with 2 mM L-alanyl-L-glutamine (GlutaMAX^TM^, Invitrogen Corp.), antibiotics (PSN; 50 μg/mL penicillin, 50 μg/mL streptomycin, 100 μg/mL neomycin [Invitrogen Corp.]), and 10% low-antibody, heat-inactivated, gamma-irradiated fetal bovine serum (FBS, Hyclone, GE, Healthcare Life Sciences, Pittsburgh, PA, USA). Vero E6 cells were incubated at 37 °C, and C6/36 cells at 28 °C, in 5% CO_2_ atmospheres within humidified incubators.

### 2.4. Inoculation of Cell Cultures with Tissue Homogenates

A total of 50 µL of LE, LT, and ST tissue homogenates was added to 3 mL of serum-free aDMEM supplemented with antibiotics and GlutaMax and filtered through a 0.45 µm pore-size syringe-tip filter (Grainger, Lake Forest, IL, USA) to remove contaminating bacteria and fungi. The resulting filtrates were then used to inoculate confluent monolayers of Vero E6 and C6/36 in 25 cm^2^ vented tissue culture flasks (Corning Inc., Corning, NY, USA) containing 5 mL of supplemented DMEM containing 10% FBS. Mock-inoculated cells were maintained in parallel with the inoculated flasks. The inoculated cells were monitored for the formation of virus-induced cytopathic effects (CPEs) using an inverted microscope with phase-contrast optics, with refeeds of the cells performed every 3 days. Aliquots of the spent cell culture media of cells displaying CPEs were collected and stored at −80 °C for follow-up analyses at a later point. Samples that did not display signs of virus-induced CPEs were maintained for 20 days post-inoculation (dpi), before being determined negative for the presence of DPV, and point samples were collected and stored at −80 °C for follow-up analyses at a later point.

### 2.5. Next-Generation Sequencing (NGS)

Unlike the previous DPV findings wherein Vero E6 cells but not C6/36 cells inoculated with skin SE homogenate [25], similar CPEs were observed in C6/36 cells but not in Vero E6 cells that had been inoculated with the LT and ST homogenates. The C6/36 cells inoculated with ST homogenate displayed more CPEs than the cells inoculated with LT homogenate. We thus assumed that the spent cell growth medium of the cells inoculated with ST homogenate contained proportionally more virions, and purified total RNA from it was obtained for analysis by NGS [19,21] as follows: after frozen archived spent cell culture media were thawed on ice, RNA was extracted from the virions in the spent growth media using a QIAamp Viral RNA Mini Kit (Qiagen, Valencia, CA, USA) according to the manufacturer’s protocol. A cDNA library was generated using a NEBNext Ultra RNA Library Prep kit (Illumina, San Diego, CA, USA) and sequenced on an Illumina NextSeq 1000 sequencer. Cell culture host sequences were removed using Kraken v2.0 [26,27], with *A. albopictus* genome sequences (GCA_001876365.2) as reference. Thereafter, de novo assembly of the remaining paired-end reads was performed using MEGAHIT v1.1.4 [28,29]. The assembled contigs were subjected to Diamond BLASTX (Diamond 2.1.8) searches against the National Center for Biotechnology Information (NCBI) non-redundant protein database using OmicsBox v1.2.

### 2.6. Phylogenetic Analyses

Maximum likelihood (ML) phylogenetic trees were constructed to assess the evolutionary relationship of CHeRI orbivirus 3–5 to other orbiviruses. ML phylogenetic trees were constructed using nucleotide and amino acid alignments of the RNA-dependent RNA polymerase (VP1), the major outer capsid protein (VP2), and the innermost sub-core capsid T2 protein (VP3), along with sequences from 35 other orbiviruses retrieved from the NCBI GenBank database. The analysis also included a sequence from St. Croix River virus (YP_052942), which served as an outgroup. Each gene was aligned individually using MAFFT implemented in Geneious Prime v2022.2.2. Maximum likelihood trees were inferred using IQ-TREE with 1000 bootstrap replicates to assess clade support [30]. The resulting trees were visualized in Interactive Tree of Life (iTOL) v7.2. [31], and bootstrap values ≥80% were considered indicative of strong clade support. Sequence identity matrices were generated from representative closely related viruses using the nucleotide sequences of these genes (i.e., *VP1*, *VP2*, *VP3*) and the amino acid sequences of their corresponding proteins with the Sequence Demarcation Tool (SDT) v1.2 [32].

## 3. Results

### 3.1. Gross Examinations

**Figure 1:**
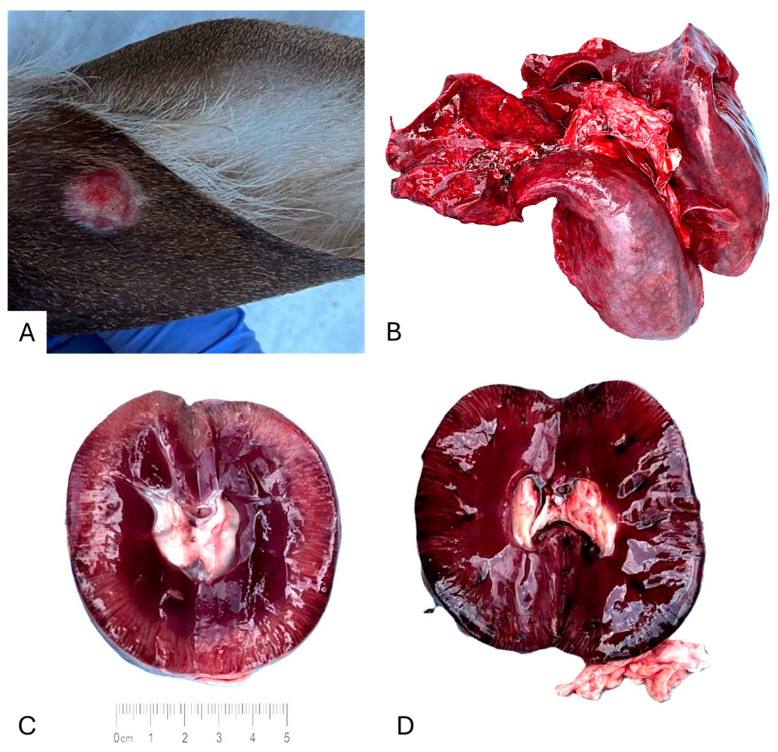
Gross observations of farmed white-tailed deer naturally infected with DPV and CHeRI orbivirus 3–5. (**A**) Ear, external view. (**B**) Lungs, external view. (**C**) Right kidney, internal view. (**D**) Left kidney, internal view.

The skin of OV1859 contained both healed and active lesions, and those findings are considered pathognomonic for DPV infection. Its liver and lungs were markedly hemorrhagic (Figure 1). The spleen was mildly pale and congested. The kidneys were grossly abnormal, one kidney appeared significantly darker than the contralateral kidney, consistent with hemorrhage; the affected kidney also exhibited abnormal tissue texture. Cardiac, rumen, and stomach tissues appeared grossly normal.

### 3.2. Cell Culture

**Figure 2:**
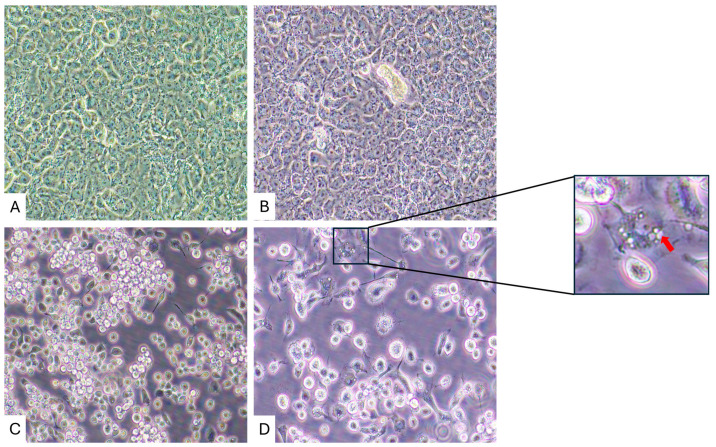
Cytopathic effects in C6/36 cells compared to no cytopathic effects in Vero E6 cells. (**A**) Mock-inoculated Vero E6 cells, 20 dpi. (**B**) Vero E6 cells inoculated with ST homogenate, 20 dpi. (**C**) Mock-inoculated C6/36 cells, 11 dpi. (**D**) C6/36 cells inoculated with ST homogenate, 11 dpi. The red arrow indicates the location of cytoplasmic inclusions. Original images were taken at 400× magnification.

Virus-induced CPEs were observed in C6/36 cells by 11 days post-inoculation (dpi) with LT and ST homogenates, but not in Vero E6 cells (Figure 2). In contrast, CPEs were not observed in Vero E6 cells inoculated with LT and ST tissue homogenates at 20 dpi. The CPEs present within the C6/36 cells included the production of cytoplasmic and peri-nuclear inclusions, as well as cell death. A representative image is shown in Figure 2.

### 3.3. Next-Generation Sequencing

Next-generation sequencing generated a total of 22,135,102 reads. Quality control and adapter trimming were performed using bcl-convert1 v4.2.4. Host-derived sequences were removed with Kraken v2.0 by mapping to the *A. albopictus* reference genome sequences (GCA_001876365.2) (1). The remaining 3,838,321 paired-end reads (17.34%) were subjected to de novo assembly. BLASTX analysis identified a CHeRI orbivirus genome comprising 10 segments with an average coverage of 9688× per nucleotide. The complete coding genome segments are available in GenBank under accession number PX208510-19 (Table 2).

### 3.4. Phylogenetic Analyses

**Figure 3:**
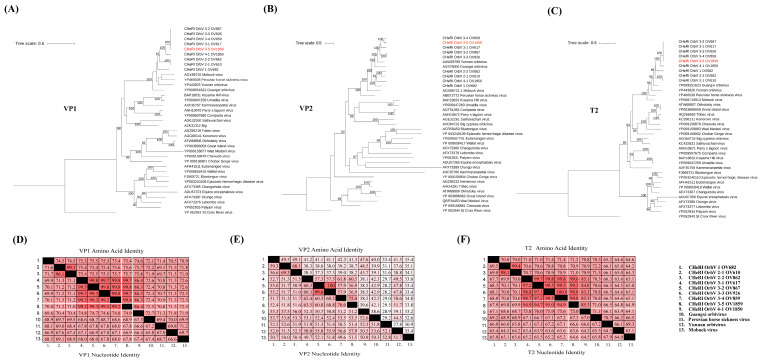
Phylogenetic and sequence identity analyses of CHeRI orbivirus 3–5. (**A**–**C**) Maximum likelihood phylogenetic trees constructed using amino acid sequences of (**A**) RNA-dependent RNA polymerase (VP1), (**B**) major outer capsid protein (VP2), and (**C**) innermost sub-core capsid T2 protein (VP3), demonstrating the relationship of CHeRI orbivirus 3–5 to other CHeRI orbivirus 3 strains (3-1, 3-2, 3-3, and 3-4). Bootstrap values (%, 1000 replicates) are indicated at each node. (**D**,**F**) Pairwise sequence identity matrices showing nucleotide (lower half) and amino acid (upper half) identities among CHeRI orbivirus 3–5 and closely related strains for (**D**) *VP1*, (**E**) *VP2*, and (**F**) *VP3* genes. CHeRI orbivirus 3–5 shares greater than 30% and 91% nucleotide and amino acid identity of *VP1* and *VP3* (T2) gene, respectively, with other CHeRI orbivirus 3 strains, whereas the *VP2* gene shows less than 74% nucleotide identity.

Phylogenetic analysis of this CHeRI orbivirus, based on amino acid sequences of the RNA-dependent RNA polymerase (VP1), major outer capsid protein (VP2), and innermost sub-core capsid T2 protein (VP3), placed it within the same clade as other strains of CHeRI orbivirus 3 (3-1, 3-2, 3-3, and 3-4) (Figure 3A–C). Sequence identity analysis of *VP1*, *VP2*, and *VP3* (T2) genes revealed that this CHeRI orbivirus shares greater than 30% (66.9–99.5%) VP1 nucleotide identity with other orbiviruses (Figure 3D), supporting its classification within the *Orbivirus* genus according to ICTV taxonomy criteria. Furthermore, the *VP3* (T2) gene exhibited greater than 91% nucleotide and amino acid identities with CHeRI orbivirus 3 strains. In contrast, the *VP2* gene showed less than 74% nucleotide identity compared to other CHeRI orbivirus 3 strains (Figure 3D,F).

## 4. Discussion

In this manuscript, we report the discovery of CHeRI orbivirus 3–5. Based on ICTV criteria for the genus Orbivirus [3], the CHeRI orbivirus identified in this study is classified within the same genus and species as previously described for the CHeRI orbivirus 3 lineage. This classification is supported by the deduced VP1 (RdRp) and VP3 (T2) amino acid sequences, which share more than 30% and 91% identity, respectively, with those of other orbiviruses. Phylogenetic analyses of VP1 and VP3 further support the close evolutionary relationship of this virus with other CHeRI orbivirus 3 strains. However, the *VP2* gene exhibited substantially lower nucleotide identity (<74%) relative to other CHeRI orbivirus 3 strains, distinguishing this virus as a novel strain within the lineage, and we have designated this virus as CHeRI orbivirus 3–5. This is the 5th CHeRI orbivirus 3 strain that has been found circulating in Florida to be associated with deaths in WTD. Ongoing genetic divergence among circulating CHeRI orbiviruses suggests active viral evolution within animal populations, necessitating monitoring of molecular diagnostics to ensure accurate reporting of disease.

The detection of CHeRI orbivirus 3–5 is novel in both the identification of a strain that has not been previously documented and this being the first instance wherein our group isolated a CHeRI orbivirus from ST in the absence of a detectable co-infecting virus. While the animal was positive for DPV, that virus was only found in skin lesion tissues and not in any internal organ samples (CT, HT, KT, LT or ST) [10]. It is possible that infection of DPV decreased the fawn’s immune function, allowing for secondary infection by CHeRI orbivirus 3–5, but the role of CHeRI orbiviruses 3–5 in disease progression or mortality cannot be determined from this case alone. Up to now, our group has only isolated CHeRI orbiviruses from ST that also contained other viruses, such as EHDV-2 and Hardee county ephemerovirus [20,21]. The isolation of CHeRI orbivirus 3–5 from the spleen of a deceased fawn without EHDV suggests a possible association between CHeRI orbiviruses and hemorrhagic disease; however, this relationship is solely based on observational evidence and animal challenge experiments must be conducted in the future to establish such casualty.

Our future goals are to understand the role of CHeRI orbiviruses in the disease of deer, as well as to identify potential intervention points for interrupting the transmission cycle of these viruses in Florida deer. The increasing number of CHeRI orbiviruses discovered in this region suggests ongoing viral diversification, potentially driven by evolutionary mechanisms such as genomic segment reassortment, which requires further investigation. Improved understanding of the ecological, evolutionary and transmission processes of CHeRI orbiviruses has important implications in the manner of which we conduct surveillance as well as our recommendations to farmers regarding best practices to prevent loss of WTD.

## Figures and Tables

**Table 1:** Results of RT-qPCR screening tests.

Virus Tested	Cq Values
BTV	>40
BVDV	>40
EHDV	>40
CHeRI 1	>40
CHeRI 2	>40
CHeRI 3	15.42
YUOV	>40
MOV	>40
BCOV	>40

**Table 2:** Inferred lengths of structural and non-structural proteins of CHeRI orbivirus 3–5.

Parameter	Segment Number and Gene Encoded ^a^									
	1	2	3	4	5	6	7	8	9	10
CDS length (bp) including stop codon	3936	2778	2613	1944	1707	1611	1326	1095	1062	750
Predicted protein length (aa)	1311	925	870	647	568	536	441	364	353	249
GenBank accession number	PX208510	PX208511	PX208512	PX208513	PX208514	PX208515	PX208516	PX208518	PX208517	PX208519

^a^ Seg. 1, *VP1* gene, encodes RdRp; Seg. 2, *VP3* gene, encodes T2 protein; Seg. 3, *VP2* gene, encodes major outer capsid protein; Seg. 4, *VP4* gene, encodes Cap; Seg. 5, *NS1* gene, encodes Tup; Seg. 6, *VP5*, encodes outer capsid protein VP5; Seg. 7, *NS2* gene, encodes ssRNA-binding protein; Seg. 8, *VP6* gene, encodes NTPase (helicase); Seg. 9, *VP7* gene, encodes immunodominant major serogroup-specific antigen; Seg. 10, *NS3* gene, encodes for protein involved in release of virus particles from infected insect cells.

## Data Availability

The amplicon sequences in this study have been deposited in the NCBI GenBank database and are available under the NCBI GenBank accession numbers PX208510-PX208519.

## Abbreviations

The following abbreviations are used in this manuscript:

aDMEM	Advanced Dulbecco’s Modified Eagle’s Medium
ATCC	American Type Culture Collection
BTV	Bluetongue virus
BVDV	Bovine viral diarrhea virus
CHeRI	Cervidae Health Research Initiative
CT	Cardiac tissue
CPEs	Cytopathic effects
dpi	Day post-inoculation
DPV	Mule deerpox virus
EHDV	Epizootic hemorrhagic disease virus
FBS	Fetal bovine serum
FS	Fecal swab
GB	GenBank
HT	Hepatic tissues
iTOL	Interactive tree of Life
KT	Kidney tissues
LE	Lesion tissue
LS	Lesion swab
LT	Lung tissue
ML	Maximum Likelihood
NCBI	National Center for Biotechnology Information
NGS	Next generation sequencing
NS	Nasal Swab
PSN	Penicillin–streptomycin–neomycin
RT-qPCR	Reverse-transcription quantitative polymerase chain reaction
SDT	Sequence demarcation tool
SK	Skin tissue
ST	Spleen tissue
WB	Whole blood
WT	White-tailed deer
